# Evaluating the Accuracy and Reliability of Real-World Digital Mobility Outcomes in Older Adults After Hip Fracture: Cross-Sectional Observational Study

**DOI:** 10.2196/67792

**Published:** 2025-05-20

**Authors:** Martin A Berge, Anisoara Paraschiv-Ionescu, Cameron Kirk, Arne Küderle, Encarna Micó-Amigo, Clemens Becker, Andrea Cereatti, Silvia Del Din, Monika Engdal, Judith Garcia-Aymerich, Karoline B Grønvik, Clint Hansen, Jeffrey M Hausdorff, Jorunn L Helbostad, Carl-Philipp Jansen, Lars Gunnar Johnsen, Jochen Klenk, Sarah Koch, Walter Maetzler, Dimitrios Megaritis, Arne Müller, Lynn Rochester, Lars Schwickert, Kristin Taraldsen, Beatrix Vereijken

**Affiliations:** 1 Department of Neuromedicine and Movement Science Norwegian University of Science and Technology Trondheim Norway; 2 Laboratory of Movement Analysis and Measurement Ecole Polytechnique Federale de Lausanne Lausanne Switzerland; 3 Translational and Clinical Research Institute Faculty of Medical Sciences Newcastle University Newcastle Upon Tyne United Kingdom; 4 Machine Learning and Data Analytics Lab Department Artificial Intelligence in Biomedical Engineering Friedrich-Alexander-Universität Erlangen-Nürnberg Erlangen Germany; 5 Geriatric Center Medical Faculty Heidelberg Heidelberg University Heidelberg Germany; 6 Department of Geriatrics and Rehabilitation Robert Bosch Hospital Stuttgart Germany; 7 Department of Electronics and Telecommunications Politecnico di Torino Turin Italy; 8 National Institute for Health and Care Research Newcastle Biomedical Research Centre Newcastle University and the Newcastle Upon Tyne Hospitals NHS Foundation Trust Newcastle Upon Tyne United Kingdom; 9 Barcelona Institute for Global Health Barcelona Spain; 10 Department of Medicine and Life Sciences Universitat Pompeu Fabra Barcelona, Catalonia Spain; 11 CIBER Epidemiología y Salud Pública Madrid Spain; 12 Department of Neurology University Hospital Schleswig-Holstein and Kiel University Kiel Germany; 13 Center for the Study of Movement, Cognition, and Mobility Neurological Institute Tel Aviv Medical Center Tel Aviv Israel; 14 Department of Physical Therapy Faculty of Medical & Health Sciences Tel Aviv University Tel Aviv Israel; 15 Sagol School of Neuroscience Tel Aviv University Tel Aviv Israel; 16 Rush Alzheimer’s Disease Center Rush University Medical Center Chicago, IL United States; 17 Department of Orthopedic Surgery Rush Medical College Rush University Chicago, IL United States; 18 Department of Orthopaedic Surgery St. Olav’s Hospital Trondheim Norway; 19 Institute of Epidemiology and Medical Biometry Ulm University Ulm Germany; 20 IB University of Health and Social Sciences Study Centre Stuttgart Stuttgart Germany; 21 Department of Sport, Exercise, and Health University Basel Basel Switzerland; 22 Department of Sport, Exercise and Rehabilitation Northumbria University Newcastle Newcastle Upon Tyne United Kingdom; 23 Novartis Biomedical Research Novartis Pharma AG Basel Switzerland; 24 Department of Rehabilitation Science and Health Technology OsloMet Oslo Norway

**Keywords:** gait, free living, digital mobility assessment, wearable device, walking speed, distance, cadence, active aging

## Abstract

**Background:**

Algorithms estimating real-world digital mobility outcomes (DMOs) are increasingly validated in healthy adults and various disease cohorts. However, their accuracy and reliability in older adults after hip fracture, who often walk slowly for short durations, is underexplored.

**Objective:**

This study examined DMO accuracy and reliability in a hip fracture cohort considering walking bout (WB) duration, physical function, days since surgery, and walking aid use.

**Methods:**

In total, 19 community-dwelling participants were real-world monitored for 2.5 hours using a lower back wearable device and a reference system combining inertial modules, distance sensors, and pressure insoles. A total of 6 DMO estimates from 164 WBs from 58% (11/19) of the participants (aged 71-90 years; assessed 32-390 days after surgery; Short Physical Performance Battery [SPPB] scores of 3-12; gait speed range 0.39-1.34 m/s) were assessed against the reference system at the WB and participant level. We stratified by WB duration (all WBs, WBs of >10 seconds, WBs of 10-30 seconds, and WBs of >30 seconds) and lower versus higher SPPB scores and observed whether days since surgery and walking aid use affected DMO accuracy and reliability.

**Results:**

Across WBs, walking speed and distance ranged from 0.25 to 1.29 m/s and from 1.7 to 436.5 m, respectively. Estimation of walking speed, cadence, stride duration, number of steps, and distance stratified by WB duration showed intraclass correlation coefficients (ICCs) ranging from 0.50 to 0.99 and mean relative errors (MREs) from –6.9% to 12.8%. Stride length estimation showed poor reliability, with ICCs ranging from 0.30 to 0.49 and MREs from 6.1% to 13.2%. Walking speed and distance ICCs in the higher–SPPB score group ranged from 0.85 to 0.99, and MREs ranged from –10.1% to –1.7%. In the lower–SPPB score group, walking speed and distance ICCs ranged from 0.17 to 0.99, and MREs ranged from 13.5% to 32.6%. There was no discernible effect of time since surgery or walking aid use.

**Conclusions:**

In total, 5 accurate and reliable real-world DMOs were identified in older adults after hip fracture: walking speed, cadence, stride duration, number of steps, and distance. Accuracy and reliability of most DMOs improved when excluding WBs of <10 seconds and were higher for WBs of >30 seconds than for WBs of 10 to 30 seconds and for participants with higher physical function. DMOs capture daily gait as early as 1 month after surgery also in people using walking aids. However, as most WBs in this cohort were short, there was a trade-off between improving accuracy and reliability by excluding short WBs and losing a substantial amount of data. These results have important implications for establishing the clinical validity of DMOs and evaluating the effects of interventions on daily-life gait, thereby facilitating the design of optimal care pathways.

## Introduction

### Background

Hip fracture after a fall is one of the most serious injuries in older adults, with a high rate of morbidity and a 1-year mortality of 22% [1,2]. Survivors of hip fracture experience a substantial decline in quality of life and mobility [3]. Mobility, the ability to move or walk around freely and easily, is essential for functioning well, maintaining independence, and ensuring social and emotional well-being [4]. Between 40% and 60% of people after hip fracture do not recover their prefracture level of mobility and ability to perform instrumental activities of daily living [3], and the incidence of nursing home admissions is high [5]. Hip fractures require surgery to restore mobility, but there is large variation in the average length of stay at the hospital across countries, ranging from 4 to 40 days after surgery [6,7]. The main goals of care in the first days are typically pain management and early mobilization, followed by rehabilitation targeting physical function and independent walking to prepare patients for returning to their daily life [8,9]. However, the different recovery trajectories and the factors determining mobility outcomes for various individuals are poorly understood. The ability to walk and regain mobility is an important indicator of recovery, making it a critical focus in the rehabilitation of patients after a hip fracture. However, getting patients back on their feet requires more than just a step in the right direction. Comprehensive mobility assessment is key to supporting effective recovery, providing valuable information about patients’ progress and informing rehabilitation strategies and optimal care pathways.

Assessing mobility requires objective, quantifiable metrics that accurately reflect a patient’s ability to move around efficiently and independently. While mobility can be described using broader measures such as upright time and number of sit-to-stand transitions [10,11], these primarily provide information about activity amount. In contrast, gait-related metrics provide information about the quality and efficiency of movement, reflecting musculoskeletal function, coordination, and stability. In addition, gait impairments are strong predictors of falls, disability, and overall health and inform about the status and progression of different health challenges [12-14], making walking a key aspect of mobility.

Gait after a hip fracture has mostly been assessed using patient-reported outcome measures that are prone to response bias or via standardized walking tests in clinical settings and laboratory assessments [15]. Traditional supervised gait assessments using sophisticated technology in laboratory settings, such as gold standard instrumented gait analysis, allow for accurate spatiotemporal measurements of gait in a controlled laboratory environment [14,16]. However, gait assessments under such conditions lack ecological validity for several reasons [17]. Laboratory assessments provide a snapshot in time, are limited by space and infrastructure, are prone to white coat effects, and often consist of isolated and structured tasks that do not necessarily reflect what people with and without mobility impairment do in their daily lives [17-19]. Importantly, they may also limit the inclusion of people from rural areas, potentially overlooking a key demographic [17]. In addition, in-laboratory assessments typically evaluate gait on even, uncluttered ground over short, straight distances only [15]. Therefore, we need more knowledge regarding gait in people’s real-world environments. In addition, there is a critical need to complement current short-term follow-up and infrequent clinical tests or assessments with more comprehensive and detailed knowledge about daily-life gait recovery throughout the first years after a hip fracture [15]. Hence, extended monitoring of gait is vital for understanding the long-term recovery process and optimizing intervention strategies [20].

However, assessing accurate gait characteristics in the real world is challenging due to internal and external confounding factors. Real-world gait is highly variable and complex as it entails navigating different walking surfaces and uneven terrain and obstacles and consists of a wide variety of related activities such as turning, stopping, and starting [21]. Methods based on the use of a single inertial measurement unit (IMU) on the lower back are available to assess gait in the real world through the quantification of digital mobility outcomes (DMOs) [20]. However, until recently, algorithms to estimate DMOs lacked comprehensive and systematic validation [22].

The Mobilise-D consortium [23] has made significant strides to change this. Mobilise-D has used a rigorous and comprehensive validation process, including a multisensor wearable setup for real-world analysis [24] and involving 1 healthy and 5 patient cohorts performing an extensive technical validation protocol [22,25]. This resulted in the identification and refinement of algorithms that enable robust gait sequence detection and subsequent estimation of key DMOs from a single inertial device worn on the lower back, such as initial foot contact, cadence, stride length, and walking speed. Previous Mobilise-D work has shown that these DMOs can be estimated accurately and reliably in the real world across a range of cohorts (healthy older adults and adults with Parkinson disease, multiple sclerosis, congestive heart failure, chronic obstructive pulmonary disease, and hip fracture), tasks, and contextual factors [26,27]. Analyses of all 6 Mobilise-D cohorts showed that the real-world algorithm performances were valid and accurate but more challenging in the case of short walking bouts (WBs; <10 seconds) and slower gait speeds (<0.5 m/s) [26,27], typical hallmarks of gait after a hip fracture. Analyses stratified by cohort indicated that the estimated walking speed and cadence in the hip fracture cohort showed moderate reliability and mean relative error (MRE) of 10% and –0.5%, respectively, and estimated stride length with poor reliability and an MRE of 11% [26]. However, these analyses did not consider hip fracture–specific factors that may affect the reliability and accuracy of the DMO estimates, which may be of critical relevance for the hip fracture cohort.

### Objectives

Building on the previous Mobilise-D publications [26,27], this study investigated additional DMOs and delved deeper into potential factors that may affect the reliability and accuracy of the real-world DMO estimates in the hip fracture cohort. As short WBs are frequent after a hip fracture [15] and walking distance can influence spatiotemporal parameters [28], we investigated whether different WB duration categories affected the estimates. Furthermore, physical functioning is associated with variations in gait parameters and is highly compromised after a hip fracture [13,29], particularly in the early phases of recovery [30], often necessitating the use of a walking aid [31].

Hence, this study investigated the following research question: can the validated Mobilise-D real-world DMOs be accurately and reliably estimated in older adults after hip fracture considering WB duration, time since surgery, level of physical function, and potential walking aid use? To answer this question, we compared 6 DMOs estimated from a single wearable device against a reference system [24,32] and investigated whether WB duration, physical function, walking aid use, and days since surgery affected the accuracy and reliability of the DMO estimates.

Given earlier results, we expected that most real-world DMOs could be estimated accurately and reliably in the hip fracture cohort and that results would improve further when excluding WBs of short duration. In addition, as shorter WBs tend to have higher errors [26,27], we expected that DMO accuracy and reliability might be lower in older adults with low physical function than in those with better physical functioning. Moreover, as gait function is especially impaired early after a hip fracture, the accuracy and reliability of DMO estimates may be lower in the earlier stages of recovery than in later stages.

## Methods

### Study Design and Participants

This multicenter observational study used data from the technical validation study (TVS) of the Innovative Medicines Initiative 2 Joint Undertaking–funded Mobilise-D project [22,23,25]. Data were collected between July 2020 and March 2022. The participants after hip fracture were recruited from the Robert Bosch Foundation for Medical Research (Germany) and Kiel University (Germany).

The participants were recruited within 13 months of surgical treatment (fixation or arthroplasty) for a low-energy fracture of the proximal femur (*International Classification of Diseases, 10th Revision,* diagnosis codes S72.0, S72.1, and S72.2), as diagnosed through x-rays of the hip and pelvis.

Participants had to be aged ≥65 years to be included. Participants were excluded if they were unable to walk 4 m independently with or without a walking aid; had a shoe size of <36 European size; had a Montreal Cognitive Assessment [33] score of ≤15; or had an occurrence of any of the following within 3 months before inclusion: myocardial infarction, hospitalization for unstable angina, stroke, coronary artery bypass graft, percutaneous coronary intervention, or implantation of a cardiac resynchronization therapy device [22]. We aimed to recruit 20 participants. Due to challenges posed by the COVID-19 pandemic, recruitment was much slower than anticipated and further hampered by us not being allowed to recruit participants during their hospital stay. As a result, we needed to almost double the recruitment period to be able to enroll 19 participants. Of these 19 participants, 6 (32%) had to be excluded because of technological issues with the pressure insoles of the reference system. Data from the reference system from another 11% (2/19) of the participants had insufficient signal quality. These 2 participants were relatively young (aged 70 and 66 years), had a very low Short Physical Performance Battery (SPPB) score of 3, and were assessed 28 and 64 days after surgery. Data from the remaining 58% (11/19) of the participants were included in the final analysis.

### Protocol and Equipment

Participants’ activities were monitored for 2.5 hours in a real-world setting within their preferred habitual environment (home, work, or community). The activities were unstructured, but to ensure sufficient variability in the collected data, participants were encouraged to complete several activities, such as rising from a chair and walking to another room; walking up and down a flight of stairs; walking to the kitchen and preparing a drink; walking outdoors (if possible for a minimum of 2 minutes); and, if walking outside, walking up and down an inclined path [22].

Gait data were collected using a single wearable device (McRoberts DynaPort MM+; 100-Hz sampling frequency, –8 g to +8 g triaxial acceleration range and 1-mg resolution, and –2000 to +2000 degrees per second triaxial gyroscope range with a 70–millidegrees per second resolution) worn at the lower back with a Velcro belt. In addition, participants were equipped with a multisensor reference system consisting of IMUs, distance sensors, and pressure insoles named Inertial Module With Distance Sensors and Pressure Insoles (sampling frequency of 100 Hz) [22,24,32,34,35] previously validated with excellent reliability in different cohorts, including hip fracture, across a complex set of different motor tests, including simulated daily activities [24]. Specifically, 2 magneto-IMUs were fixed to the instep using clips. A third IMU was attached to the lower back using Velcro. Asymmetrically positioned distance sensors were fixed above the ankles using Velcro, and 2 pressure insoles were inserted into the shoes. To ensure synchronization, time stamps of the reference system and the single wearable device were aligned (–10 to +10 ms).

### Evaluation of DMOs

The evaluation of the DMOs was based on previous Mobilise-D work that selected the top-ranked algorithms to detect gait sequences and estimate initial contact events, stride length, and cadence within identified gait sequences [27]. The best-performing cadence and stride length algorithms for hip fracture were then used to estimate walking speed [26]. The validation of this processing pipeline was based on a minimum 95% CI intraclass correlation coefficient (ICC) threshold for performance metrics (ie, sensitivity, positive predictive value, and accuracy) of at least 0.7 and a relative error of <20%, as described in the studies by Micó-Amigo et al [27] and Kirk et al [26]. All algorithms are available in the MobGap Python library (Python Software Foundation) [36].

DMOs were evaluated at a WB level. A WB was defined as a continuous walking sequence comprising a minimum of 2 consecutive strides of both feet [37]. WBs were separated by breaks of >3 seconds, and for a stride to be included, it required a duration of 0.2 to 3 seconds and a minimum length of 0.15 m [27]. These criteria were applied to generate WBs for both the single wearable device and the reference system by initially filtering the identified strides according to the stride level definition and then assembling them into final WBs by identifying breaks in the stride sequence. Final DMOs for both systems were calculated as the average value over all strides within a WB. For a rigorous comparison of DMOs at a WB level, it was essential to focus on WBs concurrently detected by both systems using a true-positive analysis approach. Accordingly, we considered WBs with a time overlap exceeding 80% of their duration as true positives, as detailed in the work by Kirk et al [26].

For each WB, 6 gait characteristics were obtained from the single wearable device and the reference system: cadence (steps per minute; the number of steps taken per minute), stride length (meters; the length of 2 consecutive steps), number of steps, stride duration (seconds) [27], walking speed (m/s) [26], and distance (meters). The walked distance was calculated by multiplying 2 validated DMOs [27]: average walking speed × WB duration.

### Variables

#### Participant Characteristics

Age, height, weight, sex, cognitive function (Montreal Cognitive Assessment), fracture type, and surgical implant were collected for all participants. Pain while walking was assessed using a visual analogue scale (from 0 to 100, where 0 is no pain and 100 is worst pain imaginable).

#### WB Duration

Given the larger single wearable device DMO errors observed in shorter WBs [26,27], we assessed whether the accuracy and reliability of DMO estimates differed when excluding WB durations of <10 seconds [38,39]. In addition, we divided WBs of >10 seconds into 2 subcategories: WBs of 10 to 30 seconds and WBs of >30 seconds. Thus, we analyzed the DMOs in 4 categories of WB duration: all WBs combined, WBs of >10 seconds, WBs of 10 to 30 seconds, and WBs of >30 seconds. This was performed at both a WB level and a participant level.

#### Physical Function

The participants’ physical function was assessed using the SPPB 7 days before or after the 2.5-hour activity monitoring. The SPPB consists of a static balance test, a 5-time chair rise test, and a 4-m walk test at a comfortable gait speed. The total SPPB score ranges from 0 to 12, where higher scores indicate better mobility capacity and a score of <8 points indicates impaired activity of daily living functions [40]. Due to the larger DMO errors in cohorts with more impaired gait and slower walking speeds [26,27], we divided participants into 2 physical function groups based on the 8-point SPPB threshold: a lower–SPPB score group consisting of participants with a total score between 0 and 7 and a higher–SPPB score group consisting of participants with a total score between 8 and 12. This threshold resulted in 2 groups of 45% (5/11) and 55% (6/11) of the participants, respectively.

#### Time Since Surgery and Walking Aid Use

Physical function and mobility typically improve gradually in the first year after a hip fracture [15,41,42]. We collected the number of days between the surgery and the 2.5-hour free-living testing date, aggregated participant DMOs, and sorted them by number of days since surgery. We also collected information on walking aid use (yes or no and type of walking aid).

### Statistical Analyses

DMOs were evaluated at a WB level to quantify DMO estimation errors (accuracy) and reliability using the validation metrics detailed in previous Mobilise-D work [26,27]. For accuracy, absolute agreement was assessed by calculating the mean error, mean absolute error, and precision (limits of agreements) [43] between DMO estimates from the single wearable device and the reference system. MREs and mean absolute relative errors (MAREs) were calculated by dividing the (absolute) errors per WB by the corresponding estimates from the reference system, expressed in percentage, as shown in the following formulas (*SWD* refers to the single wearable device, and *RS* refers to the reference system):

1. Mean error = (1 / n) × Σ from *i*=1 to n ([DMO_SWD_i_– DMO_RS_i_])
2. MRE = (1 / n) × Σ from *i*=1 to n ([(DMO_SWD_i_– DMO_RS_i_)/DMO_RS_i_] × 100)
3. Mean absolute error = (1 / n) × Σ from *i*=1 to n (|DMO_SWD_i_ – DMO_RS_i_|)
4. MARE = (1 / n) × Σ from *i*=1 to n ([|DMO_SWD_i_ – DMO_RS_i_|/DMO_RS_i_] × 100)

For reliability, the ICC(2,1) [44] was computed to evaluate how closely each of the DMOs of the 2 systems were related. ICC values of <0.5 were considered poor, ICC values between 0.5 and 0.75 were considered moderate, ICC values between 0.75 and 0.9 were considered good, and ICC values of >0.90 were considered excellent [45].

For analyses stratified by WB duration, all 6 DMOs were included. In analyses stratified by the 2 SPPB score groups, we included the 2 most relevant DMOs for clinicians and patients: walking speed [15] and distance [46].

Walking speed and distance were also evaluated at a participant level. Histograms, *Q*-*Q* plots, and the Anderson-Darling tests were used for both variables to assess normality. Given that the DMOs were not normally distributed and the relatively low number of participants, median DMO values were computed across all WBs for each participant for the single wearable device and the reference system. The median DMO values from both systems were visualized in bar graphs with IQRs.

Data preparation and visualization were conducted in MATLAB (R2022a; MathWorks), whereas statistical analyses were conducted in Stata (version 18.0; StataCorp).

### Ethical Considerations

The protocol was approved by the Faculty of Medicine of the University of Tübingen (647/2019BO2) and the Faculty of Medicine of Kiel University (D540/19). All participants gave their written informed consent. The analysis was conducted using pseudonymized data. No financial compensation was provided to participants in this study.

## Results

### Participant Characteristics

The 11 participants included in the analyses (n=6, 55% men and n=5, 45% women) were community dwelling, and their characteristics are outlined in Table 1. Notably, the study sample included older adults after hip fracture assessed between 32 and 390 days after surgery. Across the 164 WBs, walking speed and distance ranged from 0.25 to 1.29 m/s and from 1.7 to 436.5 m, respectively, as measured using the reference system, and 22% (36/164) of these WBs were of <0.5 m/s. Of the 11 included participants, 9 (82%) had ≥2 WBs of <0.5 m/s, and 2 (18%) used a walking aid during the observation period, both using a single cane or crutch. Of the 11 participants, 5 (45%) had trochanteric fractures and were treated with intramedullary nails, whereas 6 (55%) had cervical fractures, of whom 4 (67%) received a hemiprosthesis and 2 (33%) received a total prosthesis.

**Table 1:** Characteristics of individual participants and the overall sample.

	Participant												Median (IQR)
	1	2	3	4	5	6	7	8	9	10	11		
Days since surgery	32	39	60	114	141	179	193	200	244	369	390	179 (60-244)	
Age (y)	71	76	80	84	79	83	87	72	71	83	90	80 (72-84)	
Height (cm)	180	182	169	180	158	159	174	174	158	165	174	173.5 (159-180)	
Weight (kg)	78	85	66	69	54	44	71	95	53	66	96	68.5 (54-85)	
MoCA^a^ score	26	30	27	28	30	18	25	30	22	21	19	26 (21-30)	
Walking pain (0-100)	5	3	2	4	0	30	19	4	52	2	1	4 (2-19)	
SPPB^b^ score	9	4	3	9	10	7	11	10	6	12	4	9 (4-10)	
Gait speed (4MWT^c^; m/s)	0.79	0.39	0.41	0.89	0.80	0.83	1.34	1.05	0.75	1.08	0.62	0.80 (0.69-0.97)	
WBs^d^ of <0.5 m/s, n (%)^e^	2 (14)^f^	0 (0)	10 (83)^g^	3 (14)^h^	2 (7)^i^	3 (25)^g^	2 (14)^f^	3 (18)^j^	8 (23)^k^	0 (0)	3 (30)^l^	—^m^	
Sex	Male	Male	Female	Male	Female	Female	Male	Male	Female	Female	Male	—	
Fracture type	C^n^	C	C	C	T^o^	T	T	C	T	T	C	—	
Implant	H^p^	TP^q^	TP	H	Nail	Nail	Nail	H	Nail	Nail	H	—	

^a^MoCA: Montreal Cognitive Assessment.

^b^SPPB: Short Physical Performance Battery.

^c^4MWT: 4-m walk test.

^d^WB: walking bout.

^e^Number of matched WBs with a speed of <0.5 m/s as measured using the reference system.

^f^n=14.

^g^n=12.

^h^n=21.

^i^n=27.

^j^n=17.

^k^n=35.

^l^n=10.

^m^Not applicable.

^n^C: cervical.

^o^T: trochanteric.

^p^H: hemiprosthesis.

^q^TP: total prosthesis.

### WB Duration

In total, 164 WBs met our true-positive approach [26], of which 65 (39.6%) were of <10 seconds, 60 (36.6%) were between 10 and 30 seconds, and 39 (23.8%) were of >30 seconds (Table 2). Estimations of the number of steps and distance demonstrated good to excellent reliability in all WB duration categories, with ICCs exceeding 0.83, MREs ranging from –6.87% to 5.34%, and MAREs ranging from 4.96% to 19.79% (Table 3). Walking speed and stride duration showed moderate reliability for all WBs combined and for WBs of >10 seconds, with ICCs ranging from 0.64 to 0.71, MREs ranging from –5.68% to 10.35%, and MAREs ranging from 7.51% to 19.75%. Walking speed showed good reliability and less error in longer WBs of >30 seconds (ICC=0.80; MRE=5.09%; MARE=14.5%) compared to shorter WBs of 10 to 30 seconds (ICC=0.50; MRE=12.78%; MARE=20.81%). Similarly, stride duration showed good reliability and less error in WBs of >30 seconds (ICC=0.77; MRE=–4.98%; MARE=5.3%) compared to WBs of 10 to 30 seconds (ICC=0.54; MRE=–6.13%; MARE=8.94%). Cadence showed moderate to excellent reliability in all WB categories, with ICCs ranging from 0.74 to 0.98, low MREs ranging from –0.56% to –0.34%, and MAREs ranging from 1.75% to 5.62%. In contrast, stride length showed poor reliability, with ICCs ranging from 0.30 to 0.49, an overestimation in MRE varying from 6.05% to 13.23%, and MAREs ranging from 15.46% to 20.61%.

**Table 2:** The number of true-positive walking bouts (WBs) in each WB duration category for all participants in total, per Short Physical Performance Battery (SPPB) score group, and per participant sorted by days since surgery (N=164).

WB duration^a^	Number of WBs, n (%)	True-positive WBs per SPPB score group, n (%)			True-positive WBs by days since surgery, n (%)										
		Lower	Higher	32 days		39 days	60 days	114 days	141 days	179 days	193 days	200 days	244 days	369 days	390 days
All	164 (100)	70 (42.7)	94 (57.3)	14 (8.5)		1 (0.6)	12 (7.3)	21 (12.8)	27 (16.5)	12 (7.3)	14 (8.5)	17 (10.4)	35 (21.3)	1 (0.6)	10 (6.1)
>10 s	99 (60.4)	42 (25.6)	57 (34.8)	10 (6.1)		1 (0.6)	8 (4.9)	15 (9.1)	15 (9.1)	9 (5.5)	8 (4.9)	8 (4.9)	14 (8.5)	1 (0.6)	10 (6.1)
10-30 s	60 (36.6)	27 (16.5)	33 (20.1)	5 (3)		0 (0)	4 (2.4)	8 (4.9)	13 (7.9)	7 (4.3)	5 (3)	1 (0.6)	11 (6.7)	1 (0.6)	5 (3)
>30 s	39 (23.8)	15 (9.1)	24 (14.6)	5 (3)		1 (0.6)	4 (2.4)	7 (4.3)	2 (1.2)	2 (1.2)	3 (1.8)	7 (4.3)	3 (1.8)	0 (0)	5 (3)

^a^As described in the Methods section, WBs of <10 seconds (n=65) were included in the *all WBs* category and were not reported separately.

**Table 3:** Digital mobility outcome estimates from the reference system (RS) and single wearable device (SWD), mean errors and mean relative errors (MREs) with limits of agreement (LoA), mean absolute errors (MAEs) and mean absolute relative errors (MAREs), and intraclass correlation coefficients (ICC) for all walking bouts (WBs) and WBs of >10 seconds (the latter also split into WBs of 10 to 30 seconds and WBs of >30 seconds).

WB duration			RS, mean (5% quantile, 95% quantile)		SWD, mean (5% quantile, 95% quantile)		Error, mean (LoA)		MRE (%), mean (LoA)		MAE, mean (5% quantile, 95% quantile)		MARE (%), mean (5% quantile, 95% quantile)		ICC(2,1), mean (5% quantile, 95% quantile)
**Walking speed (m/s)**															
	All^a^	0.65 (0.37, 1.03)		0.69 (0.45, 0.96)		0.03 (–0.25 to 0.31)		10.35 (–50.12 to 70.82)		0.11 (0.01 to 0.33)		19.75 (1.80, 66.32)		0.67 (0.58, 0.73)^b^	
	>10 s	0.68 (0.39, 1.05)		0.71 (0.44, 1.02)		0.03 (–0.24 to 0.30)		9.75 (–53.53 to 73.03)		0.10 (0.01, 0.33)		18.32 (1.27, 66.32)		0.71 (0.62, 0.79)^b^	
	10-30 s	0.61 (0.38, 0.98)		0.66 (0.43, 0.89)		0.05 (–0.23 to 0.32)		12.78 (–56.61 to 82.16)		0.11 (0.01, 0.32)		20.81 (1.74, 65.57)		0.50 (0.32, 0.64)^b^	
	>30 s	0.77 (0.46, 1.19)		0.78 (0.45, 1.10)		0.01 (–0.25 to 0.26)		5.09 (–46.99 to 57.16)		0.09 (0.01, 0.39)		14.50 (1.11, 79.76)		0.80 (0.69, 0.88)^c^	
**Cadence (steps per min)**															
	All^a^	89.81 (64.25, 114.21)		88.52 (65.76, 110.95)		–1.29 (–21.79 to 19.21)		–0.56 (–19.50 to 18.39)		5.47 (0.15, 17.50)		5.62 (0.16, 19.43)		0.74 (0.68, 0.80)^b^	
	>10 s	88.28 (62.32, 111.14)		87.42 (62.90, 104.36)		–0.86 (–14.73 to 13.02)		–0.39 (–13.81 to 13.04)		3.58 (0.08, 12.36)		3.84 (0.08, 13.61)		0.86 (0.80, 0.89)^c^	
	10-30 s	88.60 (64.83, 110.10)		87.52 (64.83, 102.66)		–1.08 (–18.31 to 16.15)		–0.42 (–17.01 to 16.17)		4.89 (0.16, 15.30)		5.20 (0.20, 15.89)		0.75 (0.64, 0.83)^c^	
	>30 s	87.77 (58.29, 111.48)		87.26 (59.51, 108.71)		–0.52 (–6.42 to 5.39)		–0.34 (–6.45 to 5.77)		1.57 (0.02, 10.59)		1.75 (0.03, 10.36)		0.98 (0.96, 0.99)^d^	
**Stride length (m)**															
	All^a^	0.88 (0.54, 1.24)		0.93 (0.72, 1.19)		0.05 (–0.33 to 0.43)		11.42 (–49.51 to 72.35)		0.15 (0.01, 0.41)		20.40 (1.38, 64.86)		0.45 (0.34, 0.55)^e^	
	>10 s	0.91 (0.57, 1.29)		0.96 (0.72, 1.26)		0.05 (–0.33 to 0.42)		10.40 (–49.91 to 70.71)		0.14 (0.01, 0.43)		18.58 (1.03, 64.86)		0.49 (0.36, 0.61)^e^	
	10-30 s	0.83 (0.56, 1.23)		0.90 (0.71, 1.11)		0.07 (–0.30 to 0.43)		13.23 (–47.34 to 73.80)		0.14 (0.01, 0.42)		20.61 (1.28, 63.57)		0.41 (0.21, 0.57)^e^	
	>30 s	1.04 (0.60, 1.38)		1.06 (0.89, 1.31)		0.02 (–0.38 to 0.42)		6.05 (–53.63 to 65.73)		0.13 (0.01, 0.72)		15.46 (0.80, 119.91)		0.30 (0.04, 0.53)^e^	
**Number of steps**															
	All	49.77 (6.00, 266.00)		47.00 (5.00, 233.00)		–2.77 (–18.78 to 13.23)		–5.62 (–31.00 to 19.76)		3.41 (0.00, 10.00)		10.39 (0.00, 30.00)		0.99 (0.99, 1.00)^d^	
	>10 s	76.32 (13.00, 317.00)		72.10 (13.00, 306.00)		–4.22 (–24.21 to 15.77)		–5.80 (–25.88 to 14.28)		4.87 (0.00, 21.00)		8.61 (0.00, 25.81)		0.99 (0.98, 1.01)^d^	
	10-30 s	22.42 (13.00, 38.50)		20.62 (12.50, 37.00)		–1.80 (–7.52 to 3.92)		–6.87 (–30.79 to 17.06)		2.50 (0.00, 8.00)		10.98 (0.00, 29.89)		0.90 (0.79, 0.95)^c^	
	>30 s	159.26 (40.00, 479.00)		151.31 (36.00, 423.00)		–7.95 (–37.77 to 21.87)		–4.17 (–15.72 to 7.39)		8.51 (0.00, 56.00)		4.96 (0.00, 16.86)		0.99 (0.98, 1.00)^d^	
**Stride duration (s)**															
	All	1.41 (1.06, 1.88)		1.33 (1.03, 1.65)		–0.08 (–0.40 to 0.24)		–4.65 (–25.80 to 16.50)		0.12 (0.00, 0.41)		8.15 (0.15, 26.82)		0.70 (0.55, 0.79)^b^	
	>10 s	1.43 (1.09, 1.95)		1.33 (1.08, 1.65)		–0.10 (–0.41 to 0.22)		–5.68 (–24.00 to 12.64)		0.12 (0.00, 0.42)		7.51 (0.16, 24.25)		0.64 (0.41, 0.77)^b^	
	10-30 s	1.42 (1.10, 1.91)		1.32 (1.09, 1.63)		–0.10 (–0.45 to 0.24)		–6.13 (–27.08 to 14.82)		0.14 (0.01, 0.42)		8.94 (0.52, 25.02)		0.54 (0.29, 0.70)^b^	
	>30 s	1.43 (1.09, 2.07)		1.34 (1.08, 1.67)		–0.09 (–0.35 to 0.18)		–4.98 (–18.43 to 8.47)		0.09 (0.00, 0.42)		5.30 (0.04, 20.19)		0.77 (0.54, 0.88)^c^	
**Distance (m)**															
	All	25.75 (2.36, 125.62)		24.22 (2.44, 119.63)		–1.53 (–22.16 to 19.11)		4.91 (–53.08 to 62.90)		3.75 (0.06, 15.48)		19.79 (0.92, 65.29)		0.98 (0.97, 0.98)^d^	
	>10 s	40.08 (5.40, 203.72)		37.50 (5.04, 168.91)		–2.58 (–28.93 to 23.77)		3.14 (–54.18 to 60.46)		5.66 (0.07, 26.48)		17.72 (0.86, 58.11)		0.98 (0.97, 0.98)^d^	
	10-30 s	9.62 (4.84, 19.38)		9.87 (4.69, 19.41)		0.25 (–4.61 to 5.12)		5.34 (–56.44 to 67.13)		1.71 (0.05, 5.55)		19.53 (0.86, 57.10)		0.83 (0.74, 0.89)^c^	
	>30 s	86.93 (15.61, 253.43)		80.00 (20.80, 233.73)		–6.93 (–47.31 to 33.44)		–0.25 (–49.97 to 49.47)		11.74 (0.32, 59.36)		14.93 (0.78, 79.93)		0.96 (0.94, 0.98)^d^	

^a^As reported in the work by Kirk et al [26].

^b^Moderate ICC level (0.50-0.75).

^c^Good ICC level (0.75-0.90).

^d^Excellent ICC level (>0.90).

^e^Poor ICC level (<0.50).

### Physical Function

The reliability of the walking distance estimates was excellent for both SPPB groups and all WB duration categories (ICCs≥0.94) except for WBs of 10 to 30 seconds in the lower–SPPB score group (ICC=0.63), which showed moderate reliability (Table 4). Distance was overestimated in the lower SPPB–score group for all WBs (MRE=20.05%; MARE=26.83%) and WBs of >10 seconds (MRE=20.34%; MARE=26.3%), and the error was lower for longer WBs of >30 seconds (MRE=13.52%; MARE=20.43%) than for shorter WBs of 10 to 30 seconds (MRE=24.13%; MARE=29.56%). Conversely, the distance was underestimated in the higher–SPPB score group for all WBs (MRE=–6.37%; MARE=14.55%) and WBs of >10 seconds (MRE=–9.53%; MARE=11.39%), and the error was lower for WBs of >30 seconds (MRE=–8.85%; MARE=11.49%) than for WBs of 10 to 30 seconds (MRE=–10.03%; MARE=11.32%).

In the higher–SPPB score group, walking speed reliability was good for all WB duration categories (ICC≥0.85), with a slightly underestimated walking speed and MRE ranging from –1.73% to –3.59% (MARE reaching 8.62% to 11.15%). Conversely, the lower–SPPB score group showed poor walking speed reliability and higher errors for all WBs combined, WBs of >10 seconds, and WBs of 10 to 30 seconds, with ICCs ranging from 0.17 to 0.44, MREs reaching 26.58% to 32.56%, and MAREs reaching 31.22% to 35.71%. Notably, the lower–SPPB score group had a moderate walking speed reliability for WBs of >30 seconds (ICC=0.66), with an MRE of 18.96% and MARE of 23.14%.

**Table 4:** Digital mobility outcome estimates for the higher–Short Physical Performance Battery (SPPB) score (n=6) and lower–SPPB score (n=5) groups from the reference system (RS) and single wearable device (SWD), mean errors and mean relative errors (MREs) with limits of agreement (LoA), mean absolute errors (MAEs) and mean absolute relative errors (MAREs), and intraclass correlation coefficients (ICC).

WB^a^ duration and SPPB group (score)				RS, mean (5% quantile, 95% quantile)		SWD, mean (5% quantile, 95% quantile)		Error, mean (LoA)		MRE (%), mean (LoA)		MAE, mean (5% quantile, 95% quantile)		MARE (%), mean (5% quantile, 95% quantile)		ICC(2,1), mean (5% quantile, 95% quantile)
**Walking speed (m/s)**																
	**Lower (0-7)**															
		All	0.59 (0.34, 0.91)		0.70 (0.43, 0.94)		0.12 (–0.19 to 0.42)		26.58 (–49.26 to 102.41)		0.15 (0.02, 0.39)		31.30 (2.76, 95.98)		0.44 (0.14, 0.63)^b^	
		>10 s	0.56 (0.34, 0.91)		0.68 (0.43, 0.92)		0.12 (–0.18 to 0.41)		27.70 (–54.84 to 110.25)		0.14 (0.02, 0.39)		31.22 (2.76, 95.98)		0.42 (0.01, 0.64)^b^	
		10-30 s	0.53 (0.32, 0.66)		0.67 (0.42, 0.87)		0.14 (–0.16 to 0.43)		32.56 (–54.61 to 119.72)		0.16 (0.02, 0.35)		35.71 (4.02, 95.98)		0.17 (0.00, 0.42)^b^	
		>30 s	0.63 (0.34, 1.04)		0.71 (0.44, 1.05)		0.08 (–0.21 to 0.38)		18.96 (–54.21 to 92.13)		0.11 (0.01, 0.44)		23.14 (0.77, 127.33)		0.66 (0.33, 0.85)^c^	
	**Higher (8-12)**															
		All	0.70 (0.39, 1.05)		0.68 (0.48, 0.97)		–0.03 (–0.21 to 0.15)		–1.73 (–30.26 to 26.80)		0.08 (0.01, 0.19)		11.15 (1.24, 29.51)		0.86 (0.80, 0.90)^d^	
		>10 s	0.76 (0.49, 1.19)		0.72 (0.47, 1.08)		–0.04 (–0.20 to 0.12)		–3.49 (–23.62 to 16.65)		0.07 (0.01, 0.19)		8.82 (1.24, 22.25)		0.89 (0.80, 0.93)^d^	
		10-30 s	0.68 (0.44, 1.00)		0.65 (0.46, 0.89)		–0.03 (–0.18 to 0.12)		–3.41 (–23.29 to 16.47)		0.06 (0.01, 0.16)		8.62 (1.27, 22.25)		0.85 (0.73, 0.91)^d^	
		>30 s	0.86 (0.63, 1.19)		0.81 (0.65, 1.10)		–0.05 (–0.22 to 0.13)		–3.59 (–24.49 to 17.32)		0.08 (0.01, 0.21)		9.09 (1.24, 18.61)		0.87 (0.72, 0.93)^d^	
**Distance (m)**																
	**Lower (0-7)**															
		All	22.80 (1.98, 125.62)		24.31 (2.30, 130.75)		1.50 (–9.72 to 12.72)		20.05 (–49.57 to 89.67)		3.10 (0.07, 15.48)		26.83 (0.93, 93.85)		0.99 (0.99, 0.99)^e^	
		>10 s	35.68 (5.55, 125.63)		37.85 (5.75, 145.84)		2.16 (–12.14 to 16.46)		20.34 (–52.06 to 92.75)		4.57 (0.15, 20.23)		26.30 (0.86, 90.93)		0.99 (0.98, 0.99)^e^	
		10-30 s	8.79 (4.31, 16.80)		10.66 (5.04, 16.80)		1.87 (–3.29 to 7.02)		24.13 (–50.53 to 98.80)		2.31 (0.07, 6.03)		29.56 (0.86, 90.93)		0.63 (0.29, 0.81)^c^	
		>30 s	84.09 (14.35, 237.84)		86.79 (27.33, 233.73)		2.70 (–20.70 to 26.10)		13.52 (–55.05 to 82.09)		8.65 (0.32, 26.48)		20.43 (0.33, 107.91)		0.99 (0.96, 0.99)^e^	
	**Higher (8-12)**															
		All	27.94 (2.56, 139.68)		24.16 (2.49, 119.63)		–3.78 (–28.41 to 20.85)		–6.37 (–40.14 to 27.41)		4.24 (0.04, 20.05)		14.55 (0.87, 36.45)		0.97 (0.96, 0.98)^e^	
		>10 s	43.32 (5.40, 203.72)		37.25 (4.92, 168.91)		–6.07 (–36.94 to 24.80)		–9.53 (–30.31 to 11.24)		6.46 (0.06, 34.81)		11.39 (0.87, 25.97)		0.97 (0.95, 0.98)^e^	
		10-30 s	10.30 (5.29, 21.23)		9.23 (4.46, 22.02)		–1.07 (–3.66 to 1.53)		–10.03 (–30.75 to 10.69)		1.21 (0.04, 4.03)		11.32 (0.87, 28.68)		0.94 (0.80, 0.97)^e^	
		>30 s	88.71 (21.79, 253.43)		75.76 (20.80, 194.07)		–12.95 (–57.48 to 31.58)		–8.85 (–30.07 to 12.37)		13.67 (0.56, 59.36)		11.49 (0.92, 23.42)		0.96 (0.89, 0.98)^e^	

^a^WB: walking bout.

^b^Poor ICC level (<0.50).

^c^Moderate ICC level (0.50-0.75).

^d^Good ICC level (0.75-0.90).

^e^Excellent ICC level (>0.90).

### Time Since Surgery and Walking Aid Use

**Figure 1:**
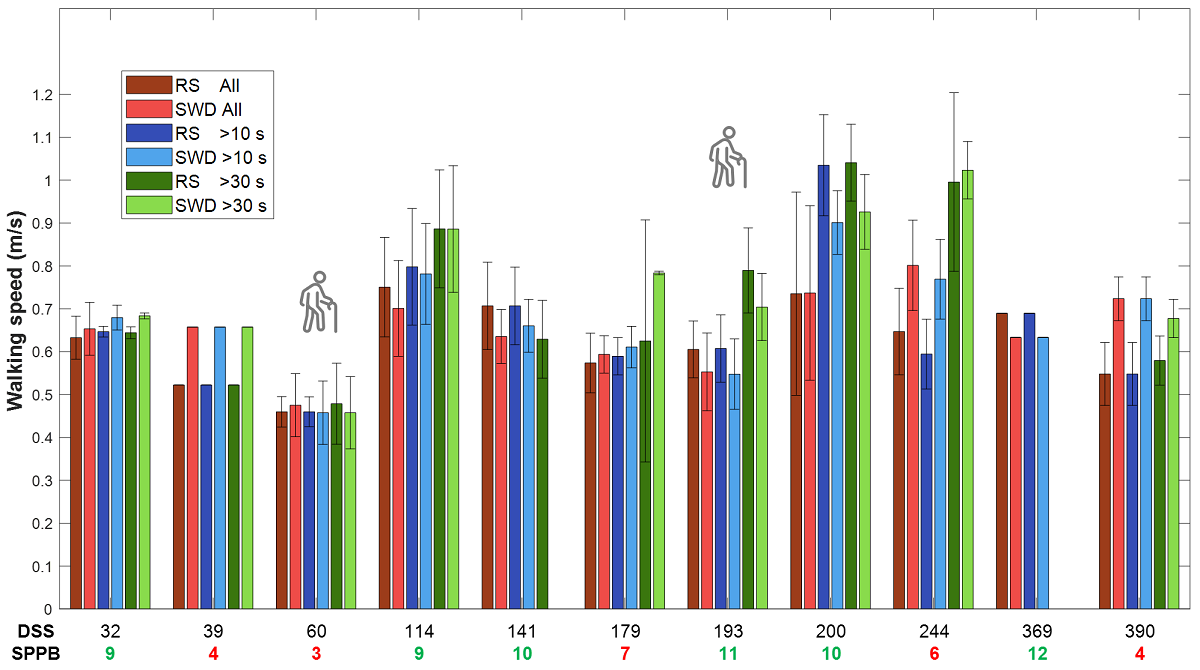
Walking speed estimates from the reference system (RS) and single wearable device (SWD) for each participant for all walking bouts (WBs) combined (red bars), WBs of >10 seconds (blue bars), and WBs of >30 seconds (green bars). The bars indicate the median, and the error bars indicate the IQR. Participants are sorted by days since surgery (DSS). The total Short Physical Performance Battery (SPPB) scores for each participant are listed below the x-axis; scores in green indicate medium or high functioning (8-12), and scores in red indicate impaired activity of daily living functions (0-7). The 2 walking aid users are marked with an icon.

**Figure 2:**
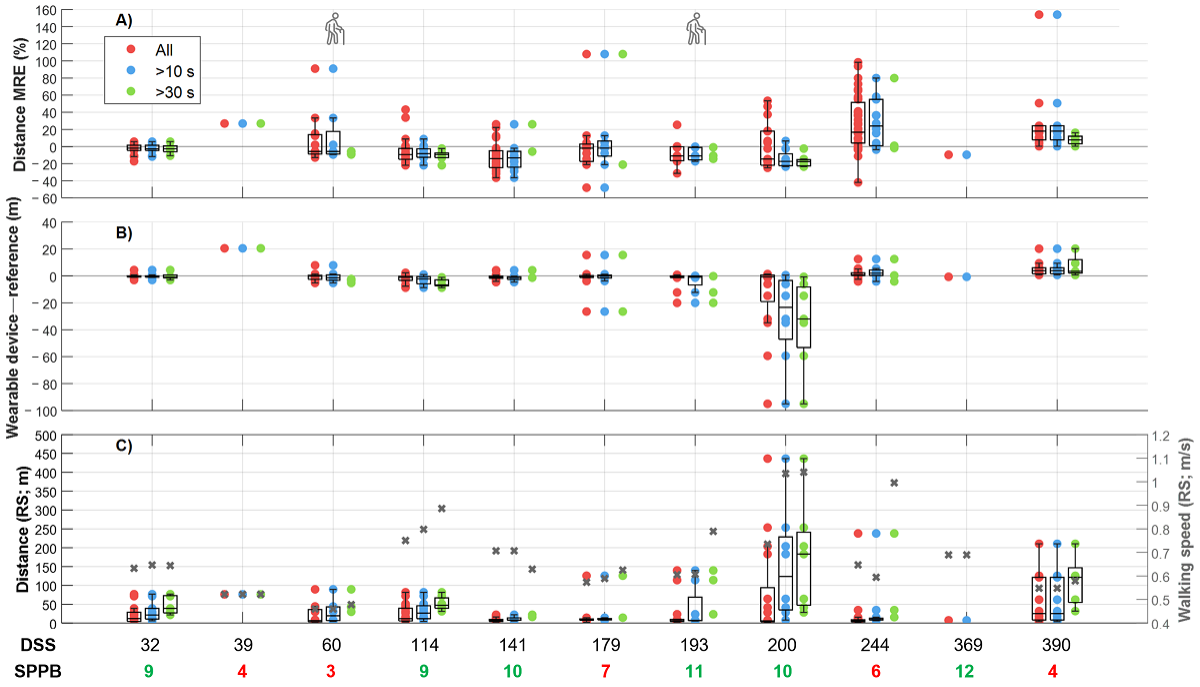
Mean relative error (MRE; A) and mean error (B) of the distance estimates from the single wearable device, and distance and median walking speed from the reference system (RS; C) for each participant for all walking bouts (WBs; red), WBs of >10 seconds (blue) and >30 seconds (green). Box plots (shown if ≥5 data points) display median, IQR, and 1.5×IQR whiskers. Participants are sorted by days since surgery (DSS). Total SPPB scores are shown below the x-axis (red: 0–7, green: 8–12). Walking aid users are marked with an icon.

To explore whether time since surgery influenced DMO accuracy, we plotted the participants’ walking speed and distance estimates from both systems sorted by the number of days since surgery, which ranged from 32 to 390. As shown in Figures 1 and 2, there was no evident pattern in DMO accuracy across the number of days since surgery for any of the WB duration categories. Similarly, no clear deviation was found for the 18% (2/11) of participants who used a walking aid.

## Discussion

### Principal Findings

This study is the first to investigate critical hip fracture–specific factors that potentially influence the accuracy and reliability of validated DMOs, building on the rigorous methods and data from the Mobilise-D TVS [22,26,27]. We compared 6 real-world DMO estimates from a single wearable device against a validated multisensor reference system [24] and investigated factors that may affect the accuracy and reliability of validated DMOs in older adults after a hip fracture: WB duration, physical function, time since surgery, and walking aid use. Overall, 5 of the DMOs (walking speed, cadence, stride duration, number of steps, and distance) showed moderate to excellent accuracy and reliability across WB durations. The sixth DMO (stride length) showed poor accuracy and reliability in this hip fracture cohort. Furthermore, walking speed and distance were more accurate and reliable in participants with better physical function. We did not observe a discernible effect of time since surgery or walking aid use on the walking speed and distance estimates. These results indicate that real-world gait assessment is feasible in a hip fracture cohort and that gait characteristics can be estimated accurately and reliably from as early as 1 month after hip fracture surgery.

### Impact of WB Duration

Previous Mobilise-D studies that validated DMOs across 6 cohorts showed generally lower algorithm performances when including very short WBs (<10 seconds) and for cohorts walking slowly and relying more on walking aids, such as many older adults after hip fracture [26,27]. As these previous studies did not perform cohort-specific analyses stratified by WB duration, this study further examined the hip fracture cohort following the true-positive evaluation of DMO accuracy and reliability at a WB level as outlined in the work by Kirk et al [26] and Micó-Amigo et al [27]. As expected, excluding shorter WBs increased the accuracy and reliability of walking speed, cadence, number of steps, stride duration, and distance, with WBs of >30 seconds having the highest accuracy and reliability. These lower errors are likely due to longer WBs capturing more continuous, consistent gait [38]. In addition, longer WB durations likely correspond to outdoor walks, which are generally not as slow and intermittent as indoor walking [38,39]. For short-duration WBs, contextual factors and turns might be more prominent [21], likely leading to higher DMO errors. However, as patients after a hip fracture typically walk in short bouts, excluding these would result in the loss of a potentially large amount of data, jeopardizing the representativeness of the data.

In addition to slow gait in short bouts, the complexity and variability of real-world environments further challenge the performance of algorithms. Factors such as deviations from a straight path, turns and obstacles, limited visibility, crowded areas, and other mobility tasks such as navigating stairs or slopes may have influenced the DMO estimates. These conditions make stride length estimation particularly challenging as this DMO is sensitive to intermittent gait [27]. Stride length exhibited larger errors and poor reliability for all WB durations, contributing to the errors in walking speed and distance estimates. This lower performance in stride length estimation could be related to the algorithms assuming an inverted pendulum, which might not properly fit the hip fracture cohort due to their asymmetrical gait [13]. Further work is necessary to improve stride length estimation in older adults after hip fracture.

### Impact of Physical Function

Our results show that walking speed can be estimated with good reliability in older adults with higher SPPB scores in all WB duration categories, with errors ranging from –3.59% to –1.73%. Conversely, the lower–SPPB score group exhibited larger errors, ranging from 26.58% to 32.56%, and demonstrated poor reliability for all WB duration categories except for WBs lasting >30 seconds, which showed moderate reliability and lower errors (18.96%). These group differences are likely due to the differences in participants’ real-world walking speed. As reported in our previous work, the performance of all algorithms decreased for speeds of <0.5 m/s [27], a threshold distinguishing between slow and medium-speed walkers [47]. Those with lower SPPB scores walked slower on average than those with higher SPPB scores (0.59, SD 0.17 m/s vs 0.70, SD 0.20 m/s, respectively), as measured using the reference system. The lower–SPPB score group walked slower and had higher errors in 10- to 30-second WBs. Although 22% (36/164) of all WBs across participants were of <0.5 m/s, only 9% (1/11) of the participants had a median speed slower than this (Figure 1 and Table 1; participant 3). Notably, this participant was among those with the lowest errors in walking speed and distance estimates despite having an SPPB score of only 3. In general, errors in slow walkers may be due to lower amplitude in the acceleration signals, inconsistent gait cycles [48,49], irregular gait patterns [50,51], short step length, shuffling gait [8], and less symmetrical gait [13]. Nevertheless, DMOs in the lower–SPPB score group demonstrated better accuracy and moderate reliability in WBs of >30 seconds, indicating that accuracy improves during longer WBs irrespective of physical function.

Patients have individual preferences regarding whether walking faster or further is more important [46], and this often depends on the context. For instance, walking speed is essential for tasks such as crossing the road, whereas walking distance is more relevant for engaging in activities such as going to the grocery store or participating in the community. As most patients report walking further as more important [46], a reliable distance estimate would provide clinicians with valuable information that is meaningful to their patients. Overall, distance estimates showed good to excellent reliability and low errors, especially for the longest WBs. When stratified by SPPB score, distance exhibited the same error pattern as walking speed, with errors being larger in shorter WBs. Although longer WBs could be more informative as these likely represent more consistent outdoor walking, for many patients, life space after a hip fracture may be confined primarily to their home environment, where furniture, walls, and doors are likely to result in significantly shorter WBs.

Distance was overestimated in older adults with lower SPPB scores and underestimated in those with higher SPPB scores, showing large variations in errors despite excellent reliability. This could be explained by the combination of overestimated average walking speed and underestimated WB duration [27], the multiplication of which yielded the distance DMO. Furthermore, high errors in the lower–SPPB score group could be explained by an inclusive WB definition that allowed for brief pauses of up to 3 seconds [37]. Such brief pauses are included in the total duration of a WB but are not taken into account in the calculation of mean walking speed, leading to a potential overestimation of distance. Older adults with lower physical function after a hip fracture may be more likely to take brief pauses and make slower turns within a WB than those with higher physical function. Therefore, when interpreting walked distance, researchers and clinicians should focus on longer and more continuous WBs if possible.

### Impact of Time Since Surgery and Walking Aids

Promisingly, our results showed no discernible change in the accuracy of walking speed and distance estimates across 32 to 390 days after hip fracture surgery, suggesting that these DMOs can be used to capture daily-life gait from as early as 1 month after surgery. As this study assessed DMO errors in the real world, we only included participants who were mobile in their home environment, which may have contributed to not having included patients within the first month after surgery. Therefore, we cannot conclude on the accuracy and reliability of DMOs in the acute phase after hip fracture. However, in the initial days and weeks following hip fracture surgery, when many patients are still in the hospital or rehabilitation center [6,7], clinical assessments may be more appropriate and informative than real-world gait monitoring. The primary focus during this period is typically first on pain management and early mobilization, subsequently followed by rehabilitation aimed at improving physical function and enabling independent walking to prepare patients for discharge to home environments [8,9]. Importantly, physical function usually improves the most during the first 3 months after hip fracture surgery [41], with DMO accuracy for participants assessed within this period showing no discernible deviation from that of participants assessed after the first 3 months. Similarly, DMO accuracy for the 18% (2/11) of participants who used walking aids did not deviate visibly from that of participants who did not. Although we found no evidence that days since surgery or the use of walking aids systematically affect DMO accuracy and reliability, these results in our small sample size should be confirmed in a larger sample.

### General Discussion

In patients after hip fracture with reduced walking ability, shorter walking duration, or slower walking speed, additional mobility outcomes may provide relevant information about their movement characteristics. The core DMOs from the Mobilise-D TVS allow for a broader assessment of additional DMOs, such as walking duration and number of WBs exceeding different duration thresholds. Other relevant metrics in this population may include the number of turns, time spent in an upright position, and the frequency of posture transitions (eg, sitting to standing). In particular, the number of sit-to-stand transitions may be an important mobility outcome for patients after hip fracture, especially in the acute phase, and ongoing research is exploring this further.

While the lower back remains a viable location for gait monitoring in the daily lives of patients after hip fracture, it may not be optimal for long-term recordings. Extended monitoring periods require sufficient battery life and may cause discomfort, particularly when sitting or lying down. In addition, adhesive attachments may irritate the skin, whereas belts used for fixation can introduce movement artifacts that compromise data quality. Other wearable device locations may help overcome some of these challenges. Although wrist-worn devices could be an option, wrist-based data are currently less accurate than lower back data [52] and have shown lower accuracy in patients recovering from hip fracture [53]. In patients with frailty and slow and cautious gait, a more suitable location for a single wearable device may be the foot or shank, which is better suited for detecting initial contact events than the lower back [54]. Furthermore, promising alternative approaches for gait monitoring in patients after hip fracture include instrumented walking aids [55] and even the prostheses themselves [56], a recent area of innovative development.

Recent comprehensive validation studies represent a significant step toward advancing real-world gait monitoring in multiple cohorts by providing robust reliability and accuracy data for DMOs [26,27]. Currently, clinically relevant accuracy thresholds have not yet been established for these DMOs in the hip fracture cohort, and they may also differ for different DMOs, making it challenging to define their appropriateness for clinical use. Our comprehensive and transparent reporting of accuracy and reliability measures allows readers to interpret the results in the context of their own research or clinical practice. Furthermore, based on the DMOs in this study, ongoing research is investigating the minimal important difference using the Mobilise-D clinical validation study dataset [57]. Further research is needed to establish clinically relevant thresholds to strengthen clinical applicability.

### Clinical Implications

As most walking activity happens in daily life, outside a laboratory or clinic, understanding gait recovery after hip fracture surgery should not only rely on snapshot tests in the clinic or assessments in the laboratory but be complemented with accurate and reliable real-world monitoring. This study shows that real-world gait monitoring can provide much-needed information about a patient’s functional recovery. Currently, the Mobilise-D consortium is undertaking a comprehensive clinical validation of all validated DMOs [57], including construct validity, predictive capacity, and the ability to detect change. Together with the encouraging results of this study, this opens up the study of real-world trajectories of gait recovery after a hip fracture and investigation of what characteristics of the patient, treatment, or rehabilitation may predict these trajectories.

This study showed that, for the hip fracture cohort, the number of steps and cadence DMOs are the most accurate and reliable, as well as suitable for both short and longer walking periods. These DMOs can also account for participants with functional constraints preventing them from performing outdoor walking. Furthermore, we found that walking speed, stride duration, and walking distance can be used to provide additional information beyond the most frequently studied DMOs, such as number of steps, cadence, and walking time [58-60]. In terms of walking speed, we recommend excluding the shortest WBs of <10 seconds to ensure accurate and reliable estimates without disregarding a disproportionate amount of data. Furthermore, clinicians should consider the patients’ physical function when interpreting walking speed due to the overestimation in people with lower SPPB scores and underestimation in people with higher SPPB scores. Distance and stride duration showed promising results across all WB durations. However, in subgroups with lower SPPB scores (<8), WBs of >30 seconds are required for obtaining more accurate distance estimates, and this may be challenging for patients with the most frailty. We showed that the distance and walking speed DMOs can be used as early as 1 month after surgery also in people using a single cane or crutch, but more data on walking aid use are needed from a larger sample.

### Strengths and Limitations

To the best of our knowledge, this is the first study to rigorously investigate the accuracy and reliability of real-world DMOs while considering critical hip fracture–related factors. A key strength is the sufficient number of 164 WBs [25], which improves the statistical power. However, the number of available WBs decreased substantially when excluding short WBs. Although this improved the accuracy and reliability of several DMOs, it also excluded a large part of participants’ data, potentially jeopardizing their representativeness of daily life activities. Moreover, some participants, especially those with better physical function, contributed more WBs than others. This imbalance may have biased the analyses by overrepresenting the walking patterns of more mobile participants. Future studies could address this by accounting for within-subject variability and ensuring more balanced data collection across participants.

While our study sample included participants with a wide range of physical function and walking speeds, with SPPB scores ranging from 3 to 12 and 21.9% (36/164) of the WBs being of <0.5 m/s, our hip fracture sample was relatively well functioning compared to the general population after hip fracture, who may have SPPB scores ranging from 1 to 5 in the first year after surgery [30]. In addition, while we had enough WBs to compare sample by sample in the true-positive analysis [25], our number of participants included in the analyses was small. As a result, formal analyses of the potential effect of days since surgery and walking aid use on DMO accuracy and reliability were not feasible.

Several factors might explain the specifics of our study sample. First, we only included participants who were mobile in their home environment. Second, as we recruited during the pandemic, we were prohibited from recruiting participants during their hospital stay, which consequently excluded those in the acute phase. Third, the complexity and thoroughness of the protocol likely also limited the recruitment of patients with the most frailty. Fourth, while we initially recruited 19 participants from a target sample size of 20, the sample size was reduced to 11 (58%) in the final analysis, primarily due to technical errors with the reference system. However, 11% (2/19) of the participants were excluded due to insufficient signal quality, which may have been related to their low physical function (SPPB scores of 3), potentially accompanied by shuffling or a very cautious gait, making it challenging to detect gait events. As a result, our findings may not fully generalize to patients with more severe impairments, and a larger sample is needed to provide stronger evidence. These limitations constrain our findings but offer a promising foundation for future research to improve the robustness and generalizability of DMOs in frail populations.

### Conclusions

Considering specific factors critical for older adults after a hip fracture, our study identified 5 accurate and reliable real-world DMO estimates from a single wearable device worn on the lower back: walking speed, cadence, stride duration, number of steps, and distance. The accuracy and reliability of most DMOs improved when excluding WBs of <10 seconds and were higher for WBs of >30 seconds than for WBs of 10 to 30 seconds and for participants with higher physical function. DMOs can capture daily gait as early as 1 month after surgery also in people using walking aids. However, as most patients after hip fracture perform WBs of short duration, there is a trade-off between more accurate and reliable walking speed and distance estimates and the disregard of substantial amounts of data. Our results add more granularity to real-world gait assessments in populations with severe gait impairments. They have important implications for future research as they can provide a significant contribution to clinical validation studies, randomized controlled trials, and descriptive studies on gait recovery after hip fracture that is meaningful for clinicians and patients alike. Finally, these results support the use of these DMOs to assess intervention effects on real-world gait in detail, thereby aiding to the design of optimal care pathways.

## Abbreviations

DMO: digital mobility outcome
ICC: intraclass correlation coefficient
IMU: inertial measurement unit
MARE: mean absolute relative error
MRE: mean relative error
SPPB: Short Physical Performance Battery
TVS: technical validation study
WB: walking bout

## Data Availability

The datasets generated or analyzed during the Mobilise-D technical validation study are available in the Zenodo repository [61].
